# Open air X-ray diffractometer for crystallography, compression, contraction, and structural phase transitions with variable temperature capabilities

**DOI:** 10.1016/j.mex.2024.102703

**Published:** 2024-04-12

**Authors:** Khan Alam

**Affiliations:** a IRC for Sustainable Energy Systems, King Fahd University of Petroleum and Minerals, Dhahran 31261, Saudi Arabia; b Department of Physics, King Fahd University of Petroleum and Minerals, Dhahran 31261, Saudi Arabia

**Keywords:** Open Air X-ray Diffractometer with Variable Temperature Capabilities, Phase change material, Molecular beam epitaxy, Variable-temperature x-ray diffraction, Structural phase transition, Biological samples, Bioengineering

## Abstract

It is a fact that materials contract or expand by changing their temperature. In a certain temperature range, the distance between atoms changes linearly in some materials whereas it changes non-linearly in other materials. X-ray diffraction (XRD) is one of the popular techniques used for understanding the crystal structure of these materials. However, XRD is mostly carried in open air at room temperature or require very expensive high vacuum set-ups and expensive temperature controllers for low temperature studies. Here we propose a design of a variable temperature X-ray diffractometer that can operate in dual modes: heating and cooling in open air. The proposed diffractometer has been used for studying structural phase transition in chromium nitride thin films. The results demonstrated not only the effectiveness of our proposed setup but also its applicability in advancing our understanding of complex material behaviors.

• A new design of a variable temperature X-ray diffractometer has been introduced in this paper, which can be used for acquiring XRD data while heating or cooling samples in open air.
• As a proof of concept, the newly designed variable temperature X-ray diffractometer is used for studying structural phase transition in CrN thin films.

## Graphical abstract

Schematic shows liquid nitrogen vapor directed on a CrN thin film during X-ray diffraction experiment.

**Figure:**
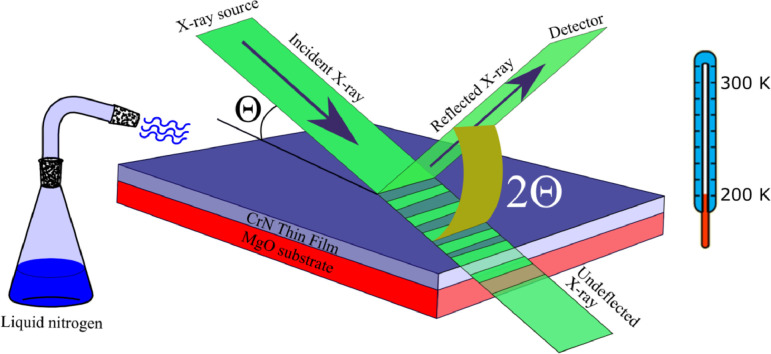

Specifications table:

**Table:** 

Subject area:	Physics and Astronomy
More specific subject area:	Condensed Matter Physics
Name of your method:	Open Air X-ray Diffractometer with Variable Temperature Capabilities
Name and reference of original method:	X-ray Diffractometer
Resource availability:	The details will be provided upon request.

## Method details

The phenomenon of materials undergoing contraction or expansion in response to temperature changes is an established fact. At the atomic scale, the behavior of various materials becomes interesting: in some cases, the distance between atoms follows a linear trend of either increasing or decreasing as temperature shifts. However, in contrast, some materials exhibit more complex behaviors, exhibiting non-linear contractions or expansions as temperature varies. From megaprojects like railways down to nanoscale transistors, thermal expansion, contraction, and phase transitions are considered. In nanotechnology, when the size of a device is in nanoscale, no material with large thermal expansion can be used. For other applications such as thermal sensors in the form a bimetallic strip, it critical to consider two different materials in which one has much larger thermal expansion compared to the other, otherwise, a bimetallic strip made of two similar materials cannot work.

Going beyond these linear and non-linear thermal responses, some materials show another layer of complexity through phase transitions [1]. These transitions encompass a spectrum of changes, including alterations in the crystal structure, electronic properties, optical properties, heat capacity, and magnetic properties of the materials [2], [3], [4], [5], [6]. What is particularly captivating is that in certain materials, these transitions are not isolated events; they can be interlinked and correlated [7], [8], [9]. A classic example of such interplay can be observed in the case of chromium nitride (CrN), which is a model system for studying coupled structural, electronic, and magnetic transitions [1,^8^,10].

Researchers use a variety of experimental techniques for understanding the inner working principles of these materials and the intricacies of their atomic arrangements, among which XRD is an indispensable technique due to its versatility, durability, and low maintenance costs. XRD allows scientists to probe the crystal structure with a high degree of precision; however, conducting XRD experiments comes with its own set of challenges. Typically, these experiments are carried out in open-air ambience at either room temperature, which is useful for study of crystallography, but cannot be used studying compression, contraction, and structural phase transitions or any other correlated electron phenomena as a function of temperatures. The variable temperature XRD demands sophisticated and expensive setups to maintain a high vacuum environment along with precise temperature control mechanisms.

Proposing a novel solution to this predicament, we introduce a new design in this article, which is a versatile X-ray diffractometer capable of operating in two distinct modes—both heating and cooling—within ordinary atmospheric conditions. This innovation eliminates the need for elaborate vacuum setups and expensive temperature regulators. By offering a more accessible and cost-effective approach, this newly conceived diffractometer opens doors to a wider range of experiments. Most of the experimental instruments used for low temperature studies do not exist in many labs around the world and are very expensive and need to be operated under vacuum condition. The proposed method is easy, inexpensive, and requires no vacuum. The proposed instrument can be employed for exploring complex physical phenomena such as thermal expansion, thermal contractions, correlated electron systems, and structural phase transition in materials of interest for advanced technological applications.

Putting this innovative design to the test, we applied it to the examination of structural phase transitions in thin films of CrN. The results demonstrated not only the effectiveness of our proposed setup but also its applicability in advancing our understanding of complex material behaviors.

A brief motivation about selecting the CrN material for the experiment is that there is huge disagreement in the published literature about the structural, electronic, and magnetic phase transitions of CrN thin films. CrN crystalizes in rock salt crystal structure with a paramagnetic (PM) phase at room temperature (RT) and undergoes a transition to an orthorhombic crystal structure with an antiferromagnetic (aFM) phase below its Néel temperature (TN ∼ 270–285 K). The proposed key driver for this structural phase transition is magnetic stresses [8]. Recent experiments on CrN thin films grown on glass and Si(001) substrates using a radio frequency sputtering system reveal an electronic transition from a semiconductor at room temperature to either a metallic or semiconductor phase, contingent on the oxygen concentration in the films [2]. At room temperature, bandgap of CrN is reported from 0.07 eV to 0.7 eV [11,12], however oxygen incorporation can increase its bandgap up to 3.56 eV [13].

In this article, an exploration of structural phase transition in CrN thin films is undertaken through temperature dependent XRD analyses spanning from 203 K to 293 K. The findings reported here validate the effective functionality of the novel low-temperature x-ray diffractometer proposed in this article. This instrument holds promise for utilization in high quality teaching and exploration in diverse fields of research.

## New experimental method

**Fig. 1:**
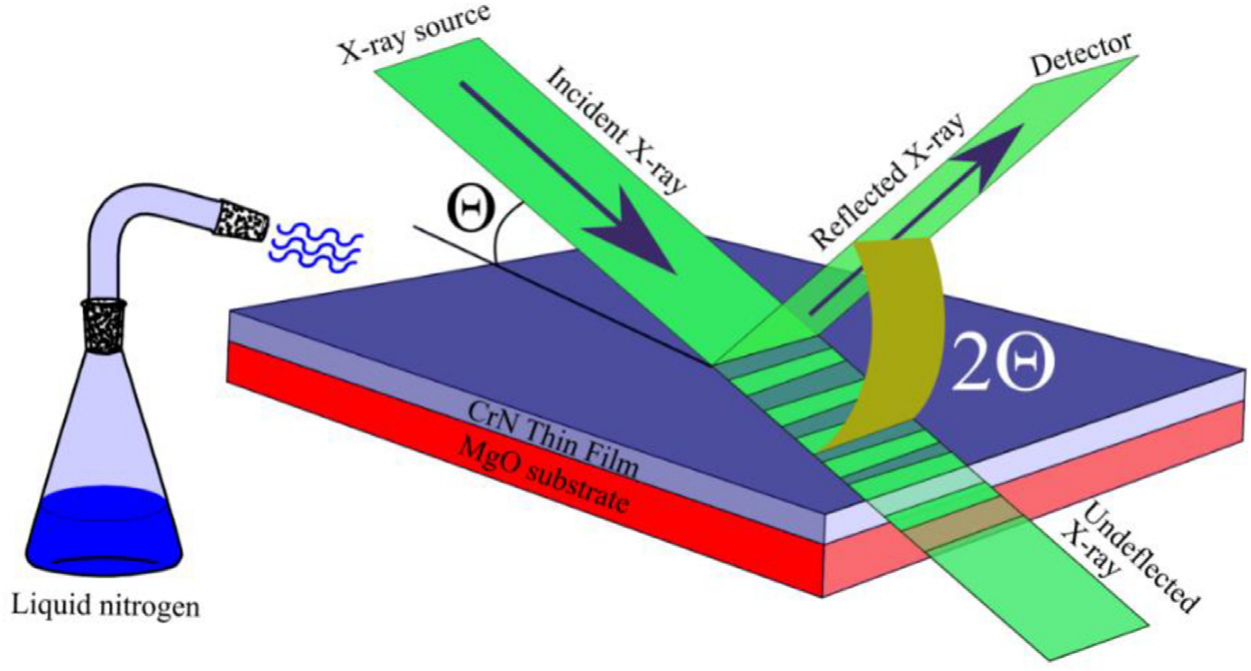
Schematic shows liquid nitrogen vapor directed on a CrN thin film during XRD experiment.

In this experiment, we custom-designed a commercially available x-ray diffractometer for variable temperature XRD measurement which was designed for a room temperature x-ray characterization of powdered samples, thin films, and biological samples. During the design process, the first thing is safety, a thermal insulation was used to the electronics and electrical wires connected to the XRD from high and low temperature sources. Moreover, the system was protected from the water vapor that condensates due to cooling. Proper water containers for collecting any droplets formed due to the condensation and water-resistant materials were used to protect the system. Only the sample holder was left open for use. We used a container for liquid nitrogen. We covered the container with an insulated layer partially and adjusted the insulated layer according to the need for cooling. For higher cooling we open the container so that room air will warm up the liquid nitrogen and the vapor coming out of the container will increase which results in more cooling of the sample and for low cooling we fully covered the container. The container had a long flexible exhaust pipe. The pipe was bent to direct the vapor on the film for cooling. The container was placed on a stable, insulating base. After filling the container with liquid nitrogen, the cold vapor will begin coming out of the container on the exhaust pipe. Since the exhaust pipe was pointed on the sample, the cold vapor will begin cooling the films. Fig. 1 shows a schematic of liquid nitrogen vapor directed on a CrN thin film during the XRD experiment. The XRD data of 002 peaks of MgO and CrN recorded in repeated mode and saved each spectrum.

The CrN thin films studied here were epitaxially grown on MgO(001) substrate. The epitaxial relationship between CrN and MgO is ${\left[100\right]}_{\text{CrN}}$||${\left[100\right]}_{\text{MgO}}$, ${\left[110\right]}_{\text{CrN}}$||${\left[110\right]}_{\text{MgO}}$, and ${\left[001\right]}_{\text{CrN}}$||${\left[001\right]}_{\text{MgO}}$ [1,^7^,14]; therefore, the 002 and 004 peaks of CrN and MgO can be seen when grown on (001) surface of an MgO substrate. The 002 peak of CrN and MgO are closer (within 2°), therefore, the temperature of the sample will be almost the same when scanning the two peaks with X-ray diffractometer. The following results show the effectiveness of the proposed method; more detailed results are reported in Ref [1]. We used the data of the linear thermal expansion of MgO to determine the temperature of the sample and used this temperature data to examine the thermal expansion of out-of-plane lattice constant of CrN. We noticed a sharp change in the out-of-plane lattice constant of CrN at the temperature which matched with the reported magneto-structural transition in CrN. The data is repeated a few times for cooling and warming cycles and every time the data was reproducible.

## Method validation

**Fig. 2:**
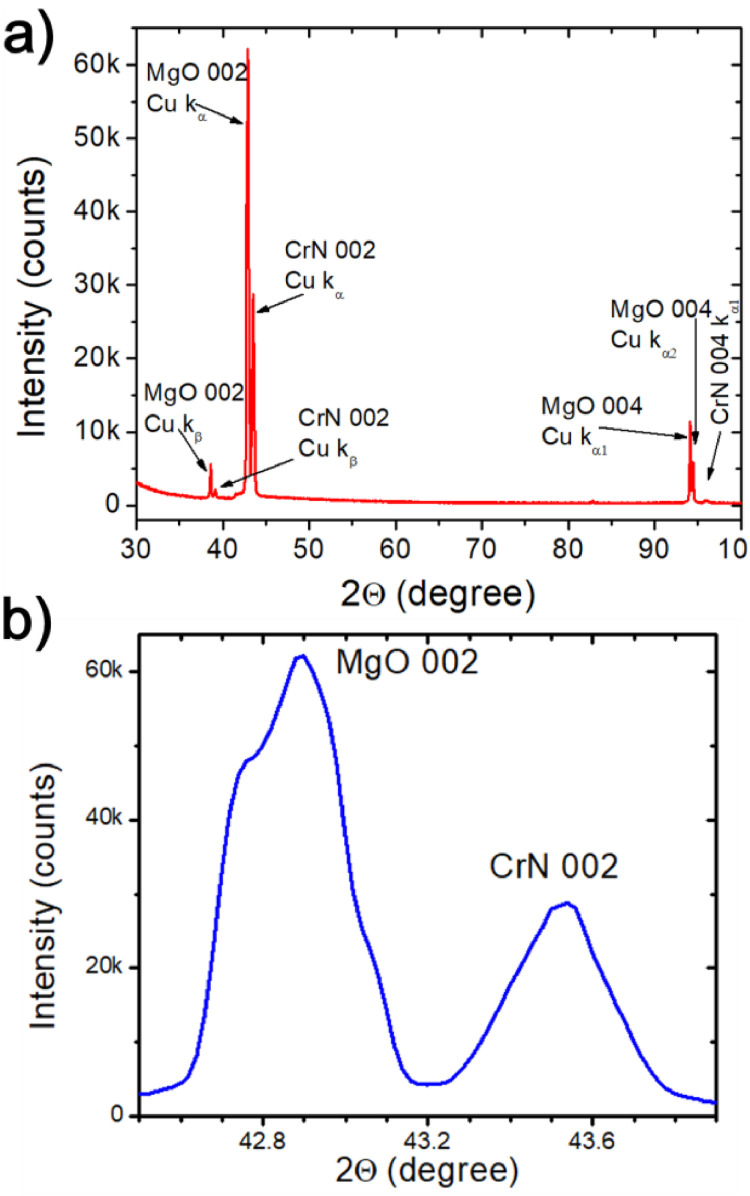
a) XRD pattern of sample CrN grown on MgO(001) substrate. b) The 002 peaks of MgO and CrN.

The XRD pattern of an epitaxially grown CrN thin film on MgO(001) substrate is shown Fig. 2(a) and a zoomed-in view of the 002 peaks of MgO and CrN is shown in Fig 2(b). The x-ray beam of a typical XRD machine consists of three wavelengths: $k_{\beta }$ = 1.3923 Å, $k_{\alpha 1}$ = 1.54059 Å, and $k_{\alpha 2}$ = 1.54439 Å. However, the intensity of $k_{\alpha 1}$ surpasses the intensities of $k_{\alpha 2}$ and $k_{\beta }$. At lower 2θ values (approximately less than 60°), the peak generated by $k_{\alpha 2}$ becomes convoluted and manifests as bulge on the right side of the peaks produced by the $k_{\alpha 1}$ wavelength. However, at higher 2θ values, $k_{\alpha 1}$ and $k_{\alpha 2}$ generate distinct peaks. The $k_{\beta }$ X-ray is more energetic than $k_{\alpha 1}$ and a typical commercial monochromator does not completely filter it out from the x-ray beam. While the peak produced by $k_{\beta }$ is negligibly small for low-intensity peaks, it becomes significant for high-intensity peaks such as the 002 peaks of MgO and CrN. The XRD measures the angle in four significant figures, which can be observed in the following section as well as in the lattice constant representation. The MgO peaks, with their corresponding wavelengths and 2θ values enclosed in parentheses, are 002 ($k_{\beta }$, 38.59°), 002 ($k_{\alpha 1}$, 42.90°), 004 ($k_{\alpha 1}$, 94.11°), and 004 ($k_{\alpha 2}$, 94.40°), whereas the CrN peaks are 002 ($k_{\beta }$, 39.19°), 002 ($k_{\alpha 1}$, 43.54°), and 004 ($k_{\alpha 1}$, 95.92°). The existence of the 002 and 004 peaks of MgO and CrN indicates that the CrN thin film is grown epitaxially on MgO(001) substrate. The lattice constant for MgO is determined to be 4.213 Å, aligning with reported literature values of 4.21 Å [15]. The 002 peak of CrN appears at 2θ = 43.54°, corresponding to an out-of-plane lattice constant $a_{⊥}$ = 4.152 Å, matches with the reported value [16]. The lattice constants for CrN and MgO in the sample are 4.213 Å and 4.152 Å, respectively. These values indicate a lattice mismatch $\frac{a_{CrN}-a_{MgO}}{a_{MgO}}\times \;100$ of 1.45%.

**Fig. 3:**
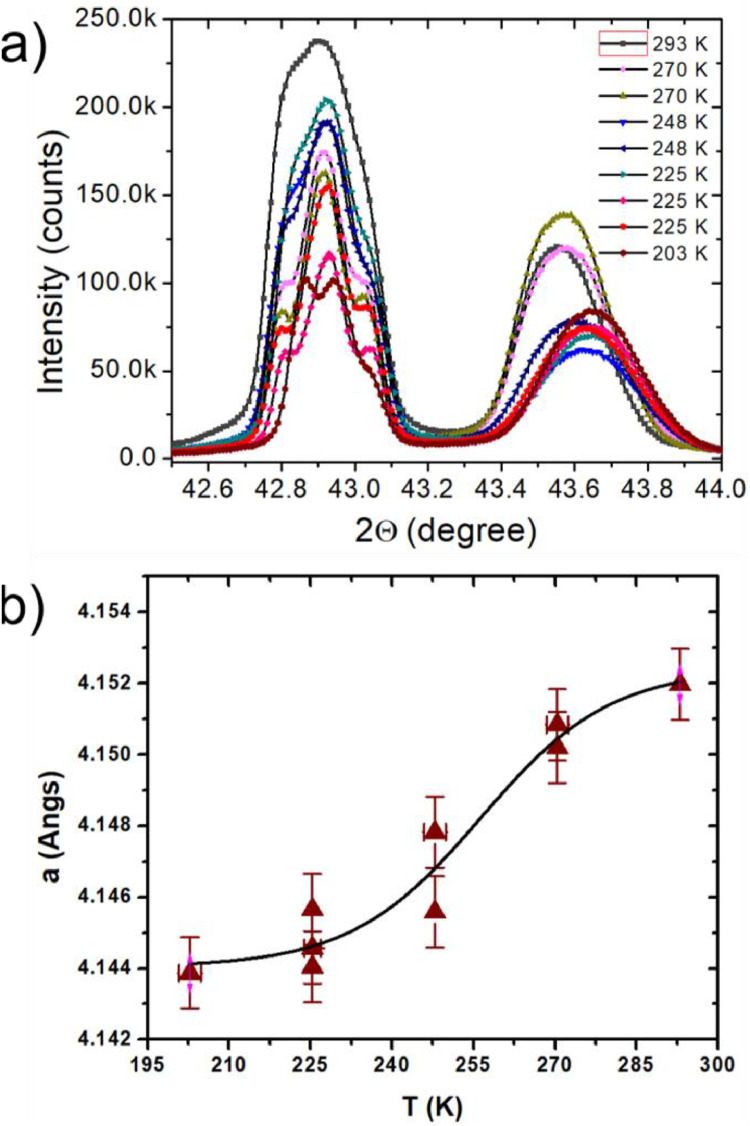
(a) The 002 peaks of MgO and CrN scanned with XRD in temperature range of 293–203 K. (b) The lattice constant of CrN exhibit structural phase transition at 256 K.

We used our custom-designed variable temperature XRD for investigating structural phase transition in CrN thin films. CrN was gradually cooled by exposing it to nitrogen vapor from room temperature to 203 K, and subsequently warmed up by controlling the vapor flow. In both the cooling and warming cycles, XRD spectra of the sample were continuously recorded. As illustrated in Fig. 3(a), during the cooling phase, the 002 peaks of MgO and CrN shifted to the right, while during warming, these peaks moved to the left, indicating contraction and expansion of the lattice constants. It is worth noting that since the experiment was not conducted under vacuum, the observed differences in intensities are likely attributed to ice formation on the surface. The MgO lattice constant exhibited linear contraction within the temperature range of the experiment, allowing for the straightforward determination of sample temperature using MgO data and the thermal expansion coefficient ($\alpha_{\text{MgO}}$) of 9.84 × 10^−6^ *K* ^−^^1^ [17,18] in equation $T\left(K\right)=\;293\;K\;+\frac{\Delta a}{a_{0}\;\alpha_{MgO}}$.

However, the CrN peak position did not exhibit a linear shift around the anticipated phase transition, as evident in the CrN out-of-plane lattice constant ($a_{⊥}$) versus temperature data presented in Fig. 3(b). A fit to the data was achieved using the Boltzmann equation [19]:

$$a_{⊥}\left(T\right)=4.152\;\overset{˚}{A}-0.008\;\overset{˚}{A}/\left[1+exp\left(\left(T-T_{N}\right)/\Delta T\right)\right],$$

This fitting yielded a transition temperature of 256 ± 6 K. On the contrary, assuming linear thermal expansion for CrN, its thermal expansion coefficient ($\alpha_{\text{CrN}}$) becomes $2.9\;\times 10^{-5}\;K^{-1}$, exceeding the reported values of $0.75-1.06\;\times 10^{-5}\;K^{-1}$ [20], [21], [22]. A similar transition in the c-axis has been reported by Zieschang et al. [23] for CrN nanoparticles.

For variable temperature analyses of other non-epitaxial samples such as nanoparticles, thin films, biological samples, and bio-engineered samples, a reference sample can be used for determining temperature of the sample. To eliminate the need for a reference sample, a temperature sensor can be installed.

## Conclusions

A new-generation, open-air x-ray diffractometer tailored for variable temperature investigation has been designed and implemented to explore structural transition in CrN thin films. This configuration offers an affordable instrument for conducting high quality variable temperature analyses of nanoparticles, thin films, biological samples, and bio-engineered samples. The sample temperature was determined using information of linear thermal expansion with no need for any temperature controller/sensors for epitaxial film, and with flexibility of installing temperature sensor for other samples. This VT-XRD can be used for studying physical phenomena like thermal contraction, expansion, and structural phase transition in materials pivotal for cutting-edge technological advancements.

## Ethics statements

No animal or social media has been used for the data collection or analysis.

## CRediT authorship contribution statement

**Khan Alam:** Conceptualization, Methodology, Validation, Data curation, Writing – original draft, Visualization, Investigation, Writing – review & editing, Funding acquisition.

## Declaration of competing interest

The authors declare that they have no known competing financial interests or personal relationships that could have appeared to influence the work reported in this paper.

## Data Availability

Data will be made available on request.
